# Evaluation of subcarinal lymph node dissection and metastasis in transmediastinal radical esophagectomy

**DOI:** 10.1007/s10388-021-00824-2

**Published:** 2021-02-18

**Authors:** Jun Shibamoto, Hitoshi Fujiwara, Hirotaka Konishi, Atsushi Shiozaki, Takuma Ohashi, Takeshi Kubota, Hiroki Shimizu, Tomohiro Arita, Yusuke Yamamoto, Ryo Morimura, Yoshiaki Kuriu, Hisashi Ikoma, Kazuma Okamoto, Eigo Otsuji

**Affiliations:** Division of Digestive Surgery, Department of Surgery, Kyoto Prefectural University of Medicine, 465 Kawaramachi Hirokoji, Kajii-cho, Kamigyo-ku, Kyoto, 602-8566 Japan

**Keywords:** Esophageal cancer, Transmediastinal esophagectomy, Subcarinal lymph node dissection, Lymph node metastasis, Predictive factor

## Abstract

**Background:**

The aim of the present study was to evaluate subcarinal lymph node dissection in transmediastinal radical esophagectomy and subcarinal lymph node metastasis in patients with esophageal cancer.

**Methods:**

Three hundred and twenty-three patients with primary esophageal cancer who underwent transmediastinal or transthoracic esophagectomy with radical two- or three-field lymph node dissection were retrospectively investigated. The clinicopathological characteristics of patients with subcarinal lymph node metastasis were analyzed in detail.

**Results:**

The median of dissected subcarinal lymph nodes in transmediastinal and transthoracic esophagectomy groups was 6 and 7, respectively, and there was no significant difference between the two groups (*p* = 0.12). Of all patients, 26 (8.0%) were pathologically diagnosed as positive for subcarinal lymph node metastasis, whereas only 7 (26.9%) of those with metastasis were preoperatively diagnosed as positive. In addition, all patients with subcarinal lymph node metastasis had other non-subcarinal lymph node metastasis. By univariate analysis, subcarinal lymph node metastasis was found in larger (≥ 30 mm) and deeper (T3/T4a) primary lesions (*p* = 0.02 and 0.02, respectively), but it was not found in 49 patients with the primary lesion located in the upper thoracic esophagus.

**Conclusions:**

Subcarinal lymph nodes can be dissected in transmediastinal esophagectomy, almost equivalent to transthoracic esophagectomy. The tumor size, depth, and location may be predictive factors for subcarinal lymph node metastasis.

## Introduction

Esophageal cancer (EC) is the eighth-most common malignancy and the sixth-leading cause of cancer-related death worldwide [1]. Although surgical techniques, postoperative management, and treatment strategies have advanced, the 5-year age-standardized survival rate of EC has not significantly improved, ranging from 10 to 30% in most countries [2]. Esophageal squamous cell carcinoma (ESCC) is the common histological type of EC in Japan and Asia. It spreads at an early stage through abundant lymphatic channels in the lamina propria mucosa and submucosa of the esophagus, and metastasizes frequently to the mediastinal lymph nodes (LNs), especially those along bilateral recurrent laryngeal nerve [3].

Subcarinal LNs, those along the tracheal bifurcation, including the bilateral main bronchial LNs, are classified as regional in ESCC, and the frequency of subcarinal LN metastasis in patients with ESCC is reported to range between 7.0 and 22.9% [4–6]. Subcarinal LN metastasis was also demonstrated to be a poor prognostic factor in patients with ESCC [4, 6, 7]. However, there are limited studies on subcarinal LN dissection and metastasis [5–7].

Esophagectomy with transcervical and transhiatal mediastinal LN dissection, transmediastinal radical esophagectomy (TME), was recently developed as radical esophagectomy without thoracotomy for EC, especially ESCC, which has the significant benefit of reducing pulmonary complications compared with transthoracic esophagectomy (TTE) [8]. TME is more safely applicable to elderly or comorbid patients, or those with difficulty in undergoing thoracotomy due to pleural adhesion or poor pulmonary function. In this procedure, subcarinal LNs are dissected by either a transcervical or transhiatal approach, or their combination.

The aim of the present study was to review subcarinal LN dissection and metastasis, and to identify the predictive factors for subcarinal LN metastasis to validate dissection.

## Materials and methods

### Patients

Between January 2008 and December 2018, 372 patients with primary EC who underwent radical esophagectomy at the Hospital of Kyoto Prefectural University of Medicine in Japan. Thirty-eight patients who underwent surgery other than TME and TTE and 11 patients with a pathologically complete response after preoperative chemotherapy were excluded, and 323 patients were enrolled. In TTE, 117 patients were performed with open surgery and 47 patients were performed with video-assisted thoracic surgery.

Clinical and pathological staging were performed using the 8th edition of the Union for International Cancer Control (UICC) tumor, nodes, and metastases (TNM) staging [9], and the 11th edition of the Japanese Classification of Esophageal Cancer (JCEC) was also used for detailed classification of regional LNs [10, 11]. Subcarinal and main bronchial LNs were classified as No. 107 and No. 109, respectively, in the JCEC, and collectively described as subcarinal LN in this study. Computed tomography (CT) and positron emission tomography (PET)-CT were performed preoperatively for the TNM staging. CT was used to evaluate treatment response before surgery for patients with preoperative treatment.

### Surgical procedure for subcarinal LN dissection

For TME, patients were placed in the supine position with both arms fixed to the trunk and both lower limbs abducted. Esophagectomy with radical lymphadenectomy was performed using transcervical and transhiatal approaches with single-port mediastinoscopy and laparoscopy. The subcarinal LNs were dissected via either a transcervical or transhiatal approach, or their combination. The details of the surgeon’s position, skin incision, port placement, and procedures are described in the previous reports [12, 13]. For TTE, patients were placed in the left lateral-decubitus position, and the thoracic procedure was performed with an open or thoracoscopic approach.

### Postoperative follow-up in the outpatient clinic

All patients were postoperatively followed up once every 3–6 months and the follow-up was continued for at least 5 years. CT or PET-CT was performed once every 4–6 months according to the patient’s condition.

### Statistical analysis

Statistical analysis was performed using JMP version 10 (SAS Institute, Cary, NC, USA). Continuous variables were indicated as medians with interquartile ranges. Fisher’s exact probability test, the chi-square test and Mann–Whitney *U* test were used to compare categorical variables between the two groups. Cancer-specific survival (CSS) was calculated using Kaplan–Meier method, with the operation date as the starting point, and differences in survival were measured using the log-rank test. All statistical tests were two-sided. *p* < 0.05 was considered significant.

## Results

### Clinicopathological characteristics

The comparison of clinicopathological characteristics between TME and TTE groups are shown in Table 1. No significant differences were found between the groups in sex, body mass index, clinical diagnosis, and tumor size, while there were significant differences in age, preoperative treatment, and histological type. The median of dissected subcarinal LNs in TME and TTE was 6 and 7, respectively, and no significant differences were found in the median of dissected subcarinal and total LNs. The median of metastatic LNs to subcarinal or other sites in the patients with subcarinal LN metastasis was 1 and 3, respectively. All patients with clinical stage IVB had supraclavicular LN metastasis.

**Table 1:** Comparison of clinicopathological characteristics between transmediastinal esophagectomy and transthoracic esophagectomy

	TME	TTE	Univariate
Variable	*n* = 159	*n* = 164	*p* value
Age (year), median (IQR)	68 (62–73)	66 (61–70)	0.03
Sex			0.78
Male	129	135	
Female	30	29	
Body mass index (kg/m^2^), median (IQR)	21.4 (19.4–23.5)	20.6 (18.7–23.1)	0.10
Preoperative treatment			< 0.01
Present	104	119	
Endoscopic submucosal dissection	17	5	
Neoadjuvant chemotherapy or chemoradiotherapy	87	114	
Absent	55	45	
Histological type^a^			0.05
Squamous cell carcinoma	151	162	
Adenocarcinoma	8	2	
Tumor location^b^			0.07
Ut	31	18	
Mt	76	94	
Lt/Ae	52	52	
Tumor size^a^ (mm), median (IQR)	37 (22–52)	40 (25–60)	0.31
Tumor depth^bc^			0.16
T 1/2/3/4a	50/26/83/0	46/21/93/4	
LN metastasis^bc^			0.44
N 0/1/2	83/65/11	74/76/14	
Stage^bc^			0.59
I/II/III/IVA/IVB	48/43/60/0/8	45/45/60/2/12	
Number of dissected LNs^a^, median (IQR)	36 (28–47)	40 (27–51)	0.17
Number of dissected subcarinal LNs^a^, median (IQR)	6 (3–10)	7 (4–11)	0.12

*TME* transmediastinal esophagectomy, *TTE* transthoracic esophagectomy, *IQR* interquartile range, *LN* lymph node, *Ut* upper thoracic esophagus, *Mt* middle thoracic esophagus, *Lt* lower thoracic esophagus, *Ae* abdominal esophagus

^a^Pathological diagnosis

^b^Clinical diagnosis

^c^According to the 8th edition of the International Union Against Cancer tumor, node, metastasis (TNM) classification system

### Predictive factors for subcarinal LN metastasis

We divided the patients into positive and negative for subcarinal LN metastasis, and analyzed relationships with the clinicopathological features. Twenty-six patients (8.0%) were diagnosed as positive for subcarinal LN metastasis. The results of univariate analysis of predictive factors for subcarinal LN metastasis are summarized in Table 2. There were no significant differences in subcarinal LN metastasis depending on age, sex, neoadjuvant chemotherapy, or histological type. Subcarinal LN metastasis was not detected in 49 patients with the primary lesion located in the upper thoracic esophagus. Subcarinal LN metastasis was found in larger (≥ 30 mm) and deeper (T3/T4a) primary lesions (*p* = 0.02 and 0.02, respectively). The total number of metastatic LNs was significantly higher in patients with subcarinal LN metastasis. More than 3 LN metastases were detected in most of these patients.

**Table 2:** Univariate analysis of predictive factors of subcarinal lymph node metastasis

		Subcarinal LN metastasis^a^		Univariate
Variable	Total (*n* = 323)	Positive (*n* = 26)	Negative (*n* = 297)	*p* value
Age (year)				0.52
< 70	205	15	190	
≥ 70	118	11	107	
Sex				0.15
Male	264	24	240	
Female	59	2	57	
Neoadjuvant chemotherapy				0.11
Present	201	20	181	
Absent	122	6	116	
Histological type^a^				0.82
Squamous cell carcinoma	313	25	288	
Adenocarcinoma	10	1	9	
Tumor location^b^				0.02
Ut	49	0	49	
Mt/Lt/Ae	274	26	248	
Tumor size^a^ (mm)				0.02
< 30	103	3	100	
≥ 30	220	23	197	
Tumor depth^b,c^				0.02
T 1/2	143	6	137	
T 3/4a	180	20	160	
Subcarinal LN metastasis^b,c^				< 0.01
Positive	24	7	17	
Negative	299	19	280	
Stage^b,c^				0.06
I/II	181	10	171	
III/IV	142	16	126	
Total number of LN metastasis^a^				< 0.01
< 3	241	6	235	
≥ 3	82	20	62	

*LN* lymph node, *Ut* upper thoracic esophagus, *Mt* middle thoracic esophagus, *Lt* lower thoracic esophagus, *Ae* abdominal esophagus

^a^Pathological diagnosis

^b^Clinical diagnosis

^c^According to the 8th edition of the International Union Against Cancer tumor, node, metastasis (TNM) classification system

In addition, detailed information of 26 patients with subcarinal LN metastasis is shown in Table 3. Of these patients, 14 patients underwent TME, whereas 12 patients underwent TTE; only 1 patient was diagnosed with adenocarcinoma; 26 had other non-subcarinal LN metastasis. In addition, 16 patients (61.5%) had LN metastasis to cervical or abdominal LNs, in addition to the thoracic LNs. Only 7 patients (26.9%) were diagnosed with subcarinal LN metastasis before surgery. Among 26 patients with subcarinal LN metastasis, 22 patients had one metastasis to either reginal LN, and 4 patients had 2 or more metastases to No.107 and either No.109.

**Table 3:** Characteristics of patients with subcarinal lymph node metastasis

Variable	*n* = 26
Age (year), median (IQR)	68 (62–71)
Esophagectomy	
TME/TTE	14/12
Subcarinal LN metastasis^a,b^	
No.107/No.109R/No.109L	11/4/7
No.107 + No.109R/No.107 + 109L/No.107 + 109R/L	1/2/1
Histological type^a^	
SCC/Adeno ca	25/1
Tumor location^c^	
Ut/Mt/Lt/Ae	0/16/9/1
Tumor size^a^ (mm), median (IQR)	45 (35–61)
Tumor depth^c,d^	
T 1/2/3/4a	3/4/18/1
Total number of LN metastasis^a^, median (IQR)	4 (3–7)
Preoperative diagnosis of subcarinal LN metastasis	
Yes/No	7/19

*TME* transmediastinal esophagectomy, *TTE* transthoracic esophagectomy, *IQR* interquartile range, *LN* lymph node, *Ut* upper thoracic esophagus, *Mt* middle thoracic esophagus, *Lt* lower thoracic esophagus, *Ae* abdominal esophagus, *SCC* squamous cell carcinoma, *Adeno ca* adenocarcinoma

^a^Pathological diagnosis

^b^According to the 11th edition of the Japanese Classification of Esophageal Cancer

^c^Clinical diagnosis

^d^According to the 8th edition of the International Union Against Cancer tumor, node, metastasis (TNM) classification system

### Cancer-specific survival according to subcarinal LN metastasis

**Fig. 1:**
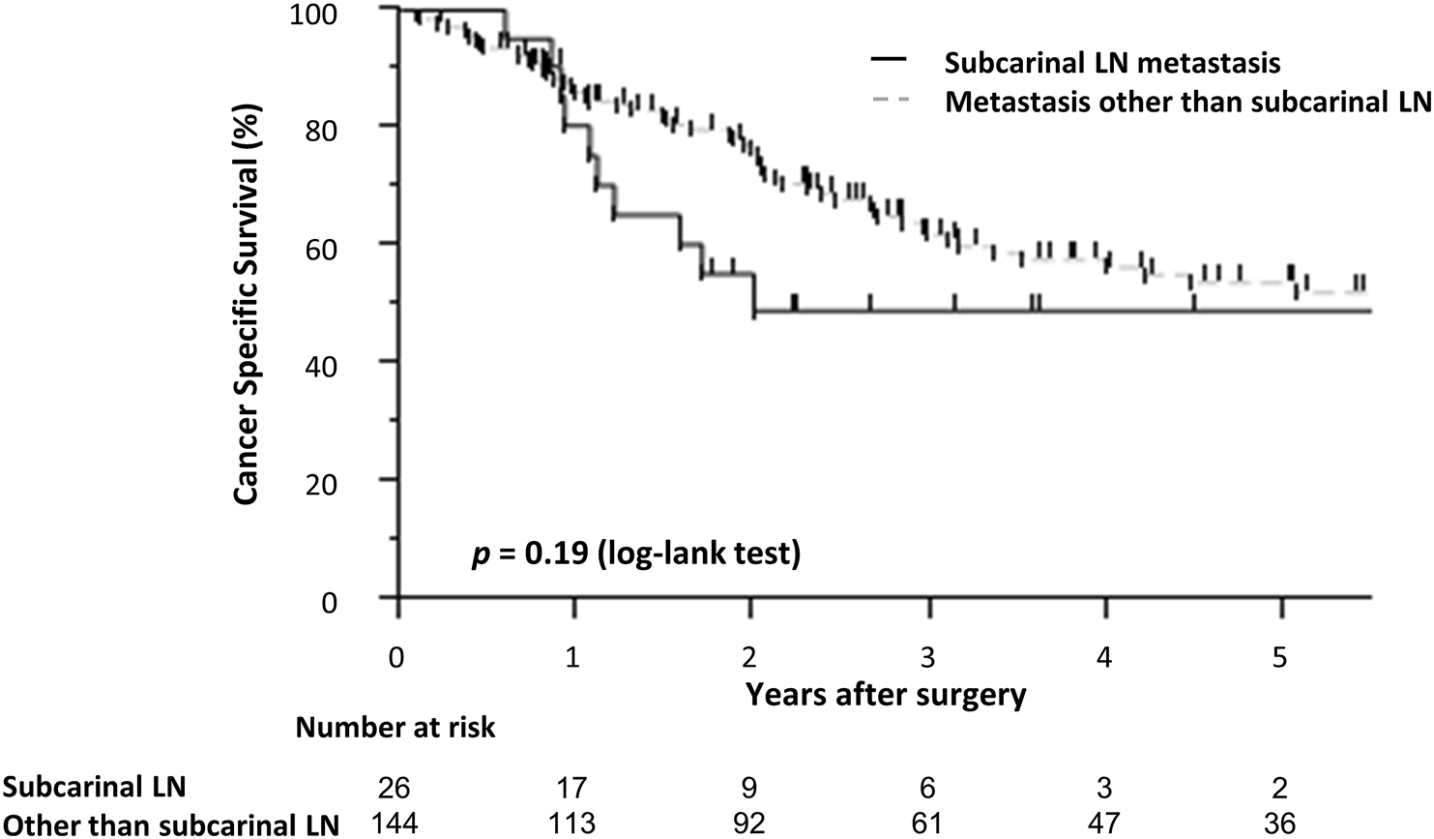
Cancer-specific survival according to subcarinal LN metastasis. Cancer-specific survival rates of patients with lymph node metastasis are shown using Kaplan–Meier method. The patients were divided into two groups, with and without subcarinal LN metastasis. The number at risk in each group is shown below the Figure. Patients who were lost to follow-up or followed up for less than 5 years were censored at the date of last contact

The cancer-specific survival was not significantly different between patients with and without subcarinal metastasis (Fig. 1). However, it was slightly poorer in patients with subcarinal LN metastasis.

## Discussion

TME was developed as an alternative surgical procedure for EC, especially ESCC, which consists of transcervical and transhiatal approaches for mediastinal LN dissection equivalent to TTE [14]. The subcarinal LNs are among those present in the deep mediastinal space, which are the most difficult to approach due to their location far from both the cervical and abdominal sides. Therefore, skill is needed for safe and careful dissection. According to the previous reports on TTE, the average or median of dissected total and subcarinal LNs ranges from 14 to 46 [15–17] and from 2 to 8 [4, 18], respectively, which was equivalent to this study with the median of dissected total and subcarinal LNs was 40 and 7, respectively. In addition, the median in TME was 36 and 6, respectively, suggesting that the transmediastinal approach yields comparable curability to the transthoracic approach in terms of the number of dissected LNs, including subcarinal LNs.

In the present study, subcarinal LN metastasis was observed in patients with more advanced disease than in those without subcarinal LN metastasis, and a longer tumor size (≥ 30 mm) and deeper tumor invasion (T3/T4a) were significant predictors for subcarinal LN metastasis. In addition, there was no subcarinal LN metastasis observed in patients with the primary tumor located in the upper thoracic esophagus. Subcarinal LN metastasis was reported to be markedly rare in patients with superficial ESCC [3, 19]. These findings may help us decide preoperatively whether to dissect subcarinal LNs considering the difficulty in making a preoperative diagnosis of LN metastasis. The diagnostic accuracy for LN metastasis in ESCC by CT or magnetic resonance imaging (MRI) is generally low, with a specificity of 51.3% and negative predictive value of 37.7% [20]. In the present study, only 26.9% (7/26) of the patients with subcarinal LN metastasis were diagnosed accurately before surgery.

Subcarinal LN dissection should be considered in relation to its prognostic impact. In this regard, subcarinal LN metastasis was demonstrated to be significantly associated with a poor prognosis [4, 6, 7]. This is consistent with our findings that patients with subcarinal LN metastasis had multiple and extensive metastases to other sites, with a slightly poorer survival than those without subcarinal LN metastasis. Subcarinal LNs may be not be the first site of metastasis and may be affected secondarily. Tachimori et al. [21] reported the prognostic significance of LN dissection in patients with thoracic ESCC using the efficacy index (EI), which is calculated by multiplying the frequency of metastasis to a specific region and the 5-year survival rate of patients with metastasis to that region. According to the study, the EI in the middle mediastinal region including subcarinal LNs is markedly lower than that in the upper mediastinal region including recurrent laryngeal nerve LNs, regardless of tumor location. Moreover, Udagawa et al. [22] and Niwa et al. [4] examined the EI of individual LN stations, and found that the EI of subcarinal LNs in patients with upper thoracic EC was lower than that in patients with middle or lower thoracic EC. In the present study, there was no subcarinal LN metastasis in patients with upper thoracic EC. In contrast, the overall survival rate of patients without subcarinal LN dissection was significantly poorer than that of those with dissection in thoracic ESCC [23]. Thus, the indication of subcarinal LN dissection should be carefully considered according to tumor stage or location and patient condition, especially in TME as subcarinal LN dissection with TME is a most difficult part due to the deep location while it is easy with TTE. If TME is selectively applied to high-risk patients who are unsuitable for thoracotomy, the skip of subcarinal LN dissection may be an option for a safe procedure.

The present study has several limitations. It was a retrospective single-center study and the study cohort was relatively small. It included patients with or without neoadjuvant chemotherapy. The period was different between TME and TTE. The effects of neoadjuvant therapy for LN metastasis were insufficient for evaluation due to the small sample size. Furthermore, there was no subcarinal LN metastasis in patients with upper thoracic tumors. Therefore, the preliminary findings of the present study should be confirmed in a larger patient cohort.

In summary, we evaluated subcarinal LN dissection and clinical features of subcarinal LN metastasis in patients with TME and TTE. Subcarinal LN dissection by TME is comparable with that by TTE in the number of dissected LNs. The tumor size, depth, and location are possible predictive factors for subcarinal LN metastasis.
